# Development of a UPLC-MS/MS method for quantifying KPT-335 (Verdinexor) in feline plasma for a study of PK

**DOI:** 10.3389/fvets.2024.1438295

**Published:** 2024-07-26

**Authors:** Yuxin Yang, Jicheng Qiu, Jingyuan Kong, Yuying Cao, Yu Liu, Sumeng Chen, Zeyu Wen, Feifei Sun, Xingyuan Cao

**Affiliations:** ^1^Department of Veterinary Pharmacology and Toxicology, College of Veterinary Medicine, China Agricultural University, Beijing, China; ^2^College of Animal Science and Technology, Anhui Agricultural University, Hefei, China

**Keywords:** UPLC-MS/MS, KPT-335, pharmacokinetics, cat, plasma

## Abstract

KPT-335 (Verdinexor) is a novel SINE that potently inhibits the nucleoprotein Exportin 1 (XPO1/CRM1) of tumor cell lines and reduces the replication level of the influenza virus. KPT-335 is mainly used for the treatment of canine tumors. Drugs for the effective treatment of feline tumors are currently unavailable in China. KPT-335 may have potential in the treatment of cat tumors. However, the effects of KPT-335 in cats are unreported, and no relevant methodology has been established for pharmacokinetic studies. In this study, a UPLC-MS/MS method was developed to determine KPT-335 concentrations in cat plasma, followed by pharmacokinetic studies. Briefly, plasma proteins are precipitated with acetonitrile, and the supernatant was collected for detection after centrifugation. The linearity for KPT-335 in cat plasma was in the range of 5–1,000 ng/mL. Satisfactory accuracy and precision were obtained. The intra-day accuracy was between −4.10% and 10.48%, the precision was ≤4.65%; the inter-day accuracy was between −0.11% and 8.09%, and the precision was ≤5.85%. Intra-day and inter-day accuracy and precision were within regulatory limits. The results of preliminary pharmacokinetic studies were as follows: T_max_ was 1.46 ± 0.51 h; C_max_ was 239.54 ± 190.60 ng·mL^−1^; T_1/2_ was 5.16 ± 2.30 h; AUC_0-t_ was 1439.85 ± 964.64 ng·mL^−1^·h. The AUC_0-∞_ was 1589.82 ± 1003.75 ng·mL^−1^·h. The purpose of this study was to develop a rapid and simple UPLC-MS/MS method to detect KPT-335 concentration in cat plasma and to conduct preliminary pharmacokinetic studies to support the future application of KPT-335 in felines.

## 1 Introduction

**Figure 1:**
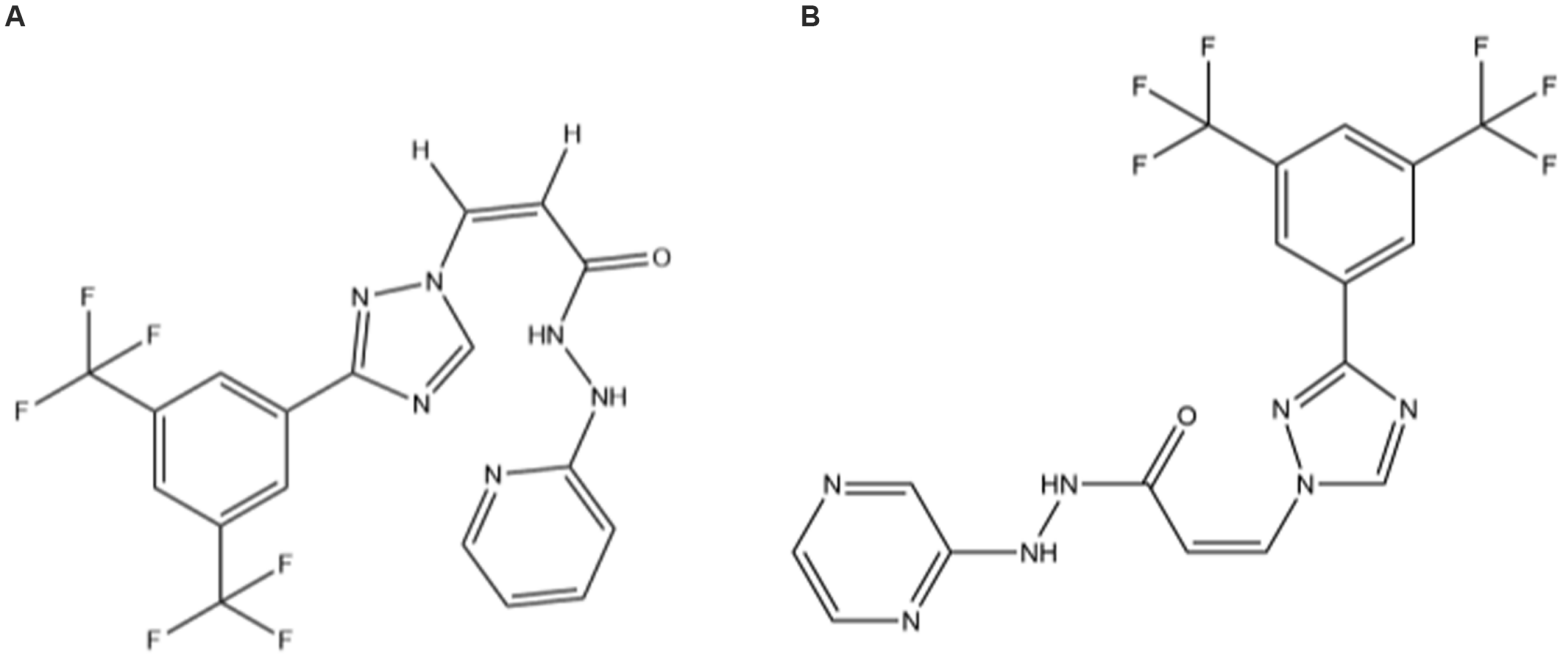
The structure of the KPT-335 **(A)** and KPT-330 **(B)**.

Pets have become indispensable in the daily lives of humans, and dog and cat diseases have gradually gained the focus of veterinarian attention. Among these, tumor diseases have developed rapidly in recent years. Soft-tissue tumors and breast tumors are among the most common tumors in cats, with a high degree of malignancy (1). Exportin-1 (XPO1) is one of seven recognized nuclear export proteins responsible for regulating the transport of substances from the nucleus to the cytoplasm, and mediates the transport of approximately 220 proteins (2–4). XPO1 has been shown to have an increased expression in several tumor cell types (5) and has been validated as a target for cancer therapeutic interventions (6). Selective inhibitors of nuclear export (SINEs) are novel anti-tumor drugs that bind covalently to the cysteine residue (Cys528) in the NES-binding groove of XPO1 (7, 8). KPT-335 (the structural formula is shown in Figure 1A) is a SINE compound with documented anti-tumor activity *in vitro* (9–11) and has also shown good anti-tumor effects in some clinical studies of cancer in dogs (12–14). Verdinexor received conditional approval from the FDA in 2019 for the treatment of lymphoma in dogs. Studies have shown that there is an increase in the expression of p53 in cats with metastatic lymphoma (15), and XPO1 acts as a nuclear export protein that transports p53 and others. This indicates that SINE has broad research prospects and cats can be selected as target animals for related research. In recent years, many novel small-molecule SINE compounds have been developed and KPT-335 is one with great potential.

The pharmacokinetic characteristics of SINE in cats remain unknown. The pharmacokinetic profiles of a drug can play a key role in understanding the dynamic changes of the drug in target animals, guiding the development and optimization of clinical and new drugs. However, the study of pharmacokinetics needs to establish a methodology to detect the concentration of drugs in samples. Most existing studies use ultra-performance liquid chromatography–tandem mass spectrometry (UPLC-MS/MS) to detect the concentration of KPT-330 (Selinexor, a KPT-335 homolog, Figure 1B) in human, rat, or mouse plasma (16–19). There is a lack of detection methods for KPT-335 in cat plasma. The purpose of this study was to develop a rapid and simple UPLC-MS/MS method for the detection of KPT-335 concentration in cat plasma, to make preliminarily explorations of the pharmacokinetic profiles, and to provide theoretical and data support for the future application of KPT-335 in felines.

## 2 Methods

### 2.1 Chemicals and animals

KPT-335 was supplied by Shanghai Macklin Biochemical Technology Co., Ltd. (purity, 99.846%). KPT-330 was used as an internal standard (IS) and supplied by Abmole Bioscience Co., Ltd. (purity, >99%). All reagents used in the test are chromatographic or mass spectrometry grade. Six domestic short-haired cats provided by the Experimental Animal Center of China Agricultural University (No. 11305-23-E-002) were enrolled in this study. Half of the cats were male and half were female, they were aged 2–3 years, and weighed between 2.25 and 2.75 kg.

### 2.2 Instrument and conditions

For ultra-performance liquid chromatograph (UPLC), we used an Agilent 1,290 II/Prime UPLC with a Phenomenex Kinetex C18, 2.1 × 50 mm, 2.6 μm column. The mobile phase included the following: A, 0.1% formic acid aqueous solution; B, 0.1% formic acid acetonitrile solution; flow rate, 0.3 mL/min; column temperature, 40°C. The elution procedure was as follows: 90–5% A (0.5–2.5 min), 5% A (2.5–4.5 min) and returned to the initial percentage (4.5–4.6 min) and then maintained at 90% A (4.6–6.0 min).

For mass spectrometry, we used an Agilent 6,470-LC-TQ; ion source: electrospray ion source; scanning method: positive ion scanning; detection method: multi-reaction monitoring; ion source temperature: 300°C; solvent removal temperature: 300°C; capillary voltage: 3.0 kV; solvent gas flow rate: 300 L/h, the compound-related parameters in the mass spectrometry are shown in Supplementary Table S1.

### 2.3 Preparation of stock and samples

KPT-335 and KPT-330 were dissolved in methanol separately into a stock solution of 1 mg/mL. The standard stock solutions were serially diluted with methanol to obtain KPT-335 with concentrations of 10,000.0, 5000.0, 2000.0, 1000.0, 500.0, 200.0, and 50.0. The quality control stock solution was serially diluted with methanol to obtain a series of quality control working solutions at concentrations of 8000.0, 4000.0, 100.0, and 50.0 ng/mL. The IS stock solution was diluted with methanol to obtain an IS working solution with a concentration of 4000.0 ng/mL. The stock solutions were stored at −20°C and the working solutions were stored at 4°C.

Subsequently, 10 μL of the working solution was added to 90 μL plasma to obtain the calibration standard samples (concentrations of 5, 20, 50, 100, 200, 500, and 1,000 ng/mL) and quality control samples (concentrations of 5, 10, 400, and 800 ng/mL for low limit of quantification, low, medium, and high levels of quality control).

### 2.4 Sample processing

A protein precipitation method was used to process samples. A 100 μL aliquot of plasma sample was accurately pipetted into a 1.5 mL microcentrifuge tube, followed by the addition of 10 μL of IS working solution. The mixture was vortexed for 1 min, 900 μL of acetonitrile was added, then vortexed for 2 min, followed by centrifuging at 13,400 × *g* for 10 min. After centrifuging, the supernatant was filtered using a syringe filter (0.22 μm) and transferred into a 2 mL glass vial. The glass vial was placed inside the autosampler tray and 2.0 μL of sample was injected into the system.

### 2.5 Method validation

We referred to FDA, EMA, and ICH M10 guidelines for method validation, including selectivity, matrix effects, recovery, calibration curves, accuracy and precision, carryover, dilution reliability, and stability.

#### 2.5.1 Selectivity

Six blank samples of cat plasma from different sources were analyzed to observe whether there were interference signals in the regions where KPT-335 and IS peaks appeared.

#### 2.5.2 Linearity

The calibration range was 5–1,000 ng/mL, including seven calibration standards. To draw the matrix standard curve equation and correlation coefficient (*R*^2^), the ratio of the peak area of KPT-335 to KPT-330 was taken as the ordinate, and the ratio of the concentration to the theoretical value of KPT-330 concentration was used as the abscissa. The 1/X^2^ weight was selected for analysis, and an *R*^2^ should greater than 0.99.

#### 2.5.3 Precision and accuracy

Accuracy and precision are expressed as standard deviation and coefficient of variation, respectively. Four concentrations were selected for investigation: the lowest limit of quantification (LLOQ, 5 ng/mL), low (LQC, 10 ng/mL), medium (MQC, 400 ng/mL), and high (HQC, 800 ng/mL). Each concentration was in six parallels for 3 days and the intra- and inter-day standard deviations and coefficient of variation were calculated.

#### 2.5.4 Carryover

Carryover was investigated after the upper limit of quantification (ULOQ, 1,000 ng/mL) samples were performed in double blank samples.

#### 2.5.5 Extraction recovery and matrix effect

Extraction recoveries were evaluated by contrasting the peak area responses of KPT-335 at three QC concentrations. These were spiked before extraction and contrasted with those spiked after extraction, over six replicates. Some samples were prepared as QC samples and processed through sample processing. An appropriate amount of blank plasma was taken and processed according to the sample processing method. The supernatant was obtained and the working solution and IS were added. The recovery and matrix effect sample was obtained by vortexing for 1 min.

The blank plasma was replaced with 90% acetonitrile and the steps of the recovery and matrix effect sample were followed to obtain a pure solution sample. Two concentrations of LQC and HQC were investigated and six parallel samples were analyzed for each concentration.

#### 2.5.6 Dilution effect

KPT-335 samples containing concentrations higher than ULOQ were subjected to a single 10-fold dilution and three consecutive 10-fold dilutions with blank plasma. Five parallel samples were prepared for each dilution factor sample.

#### 2.5.7 Stability

KPT-335 working solution was added to blank plasma, LQC, MQC, and HQC samples to investigate the room-temperature stability for 2 h, freeze–thaw stability with three times, autosampler stability for 24 h, and long-term stability of 20 days at −20°C. The stability of the stock solution for 20 days was also investigated.

#### 2.5.8 Optimized room temperature stability

In a previous study, KPT-335 demonstrated unsatisfactory recovery results in neutral or alkaline solutions at room temperature. Given that plasma is mostly neutral or alkaline, to optimize the stability of KPT-335 at room temperature and ensure the desirable result of the sample during the detection process, we carried out further experiments. There were four groups in the experiment. The first group was the normal preparation of the sample, and the second, third, and fourth groups were samples containing 0.01, 0.05, or 0.1% formic acid, respectively. All the samples were placed at room temperature for 12 h and then processed at the same time for detection.

#### 2.5.9 Reinjection reproducibility

Preparing a set of calibration samples and quality control samples (six parallels at each concentration) at low, medium, and high concentrations, and measured immediately after the first day of sample processing; store in an autosampler tray at 8°C for 24 h and re-inject the sample.

### 2.6 Pharmacokinetic study

The recommended dose of KPT-335 for dogs is 1.5 mg/kg body weight (BW). To calculate the dose for cats, the average weight of the tested cats was substituted in the equation used for dogs, and the recommended dose was 2 mg/kg BW through body surface area (BSA) conversion (20). The formula was as follows:


(1) $$K_{m}=100÷k\times W^{0.33}$$

(2) $$\mathrm{Dosag}e_{\mathrm{cat}}=\mathrm{Dosag}e_{\mathrm{dog}}\times K_{m\;\mathrm{dog}}÷K_{m\;\mathrm{cat}}$$

where K is a constant determined for different animal species, such as a dog (11.0) and cat (8.7), and W is the BW (kg) of the animal. K_m_ is approximated by the individual animal species taking the ratio between the mean weight (kg) and BSA (m^2^), this value can be calculated using Formula 1. The average weight of the six tested cats was used in BW parameters, the dosage was calculated by Formula 2, and KPT-335 was weighed into capsules and administered to the animals. The cats were fasted for 12 h before and 2 h following drug administration. Potential adverse effects were monitored during the study. The capsules were administered orally as a single bolus to the back of the tongue via a dosing syringe, accompanied by 3–5 mL of water.

Samples of blood (1 mL) were collected from the brachial cephalic vein at 0.167, 0.333, 0.5, 0.75, 1, 1.5, 2, 4, 6, 8, 12, 24, 36, and 48 h pre- and post-oral dosing into an anticoagulant blood collection tube containig heparin sodium. Blood samples were centrifuged for 8 min at 1800 × *g*. The plasma layer was taken and divided evenly into two. The plasma samples were stored at −20°C until analysis (within 20 days of collection). For the pharmacokinetic parameters, the blood drug concentration-time data was analyzed by using WinNonlin software (version 8.1, United States). The elimination half-life (T_1/2_), the maximum plasma concentration of KPT-335 (C_max_), the time when the plasma concentration reached a maximum (T_max_), and the area under the concentration-time curve (AUC) were determined.

## 3 Results

### 3.1 Method validation

#### 3.1.1 Selectivity

**Figure 2:**
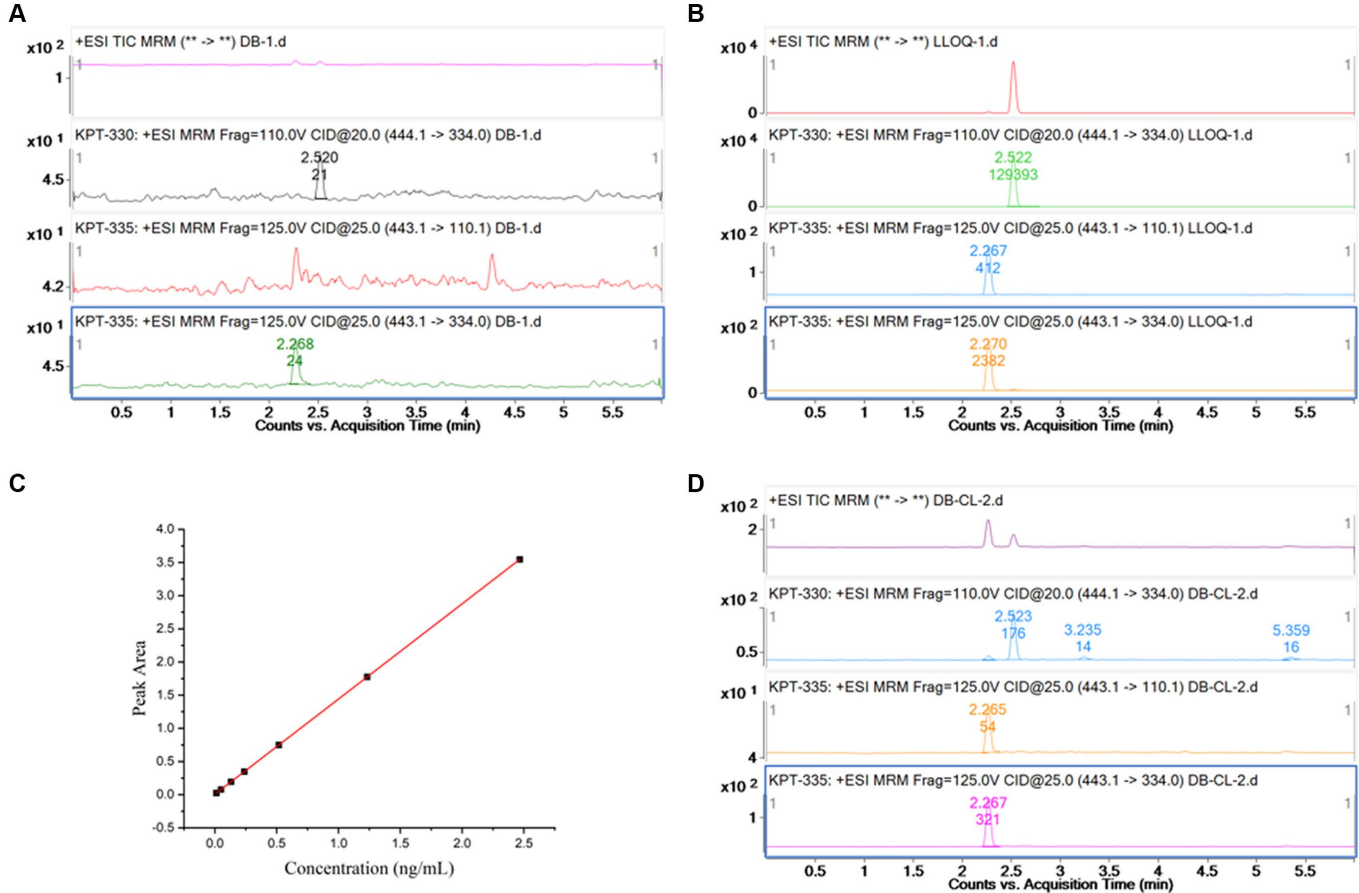
**(A)** Chromatogram for blank plasma sample; **(B)** chromatogram for KPT-335 standard substance,5 ng/mL (LLOQ); **(C)** standard curve of KPT-335 in plasma; **(D)** chromatogram for carryover sample.

As shown in Figures 2A,B, no interference peak could be observed at the retention times of KPT-335 and IS in blank samples. Its response was within 20% of the LLOQ and 5% of the IS.

#### 3.1.2 Linearity

To perform linear regression from the correlation between the concentration of the calibration standard and the ratio of the peak area of KPT-335 to the IS and to obtain the standard curve and linear equation, the least squares method was used with a weight of 1/X^2^. Plasma concentrations for KPT-335 ranged from 5 to 1,000 ng/mL. The linear regression equations with the correlation coefficients were Y = 1.30 ± 0.12X + 0.0014 ± 0.0042, *R*^2^ = 0.998, and the linear relationship was good. The standard curve is shown in Figure 2C.

#### 3.1.3 Precision and accuracy

The intra- and inter-day precision and accuracy data were analyzed at three QC concentrations and LLOQ levels in six replicates. The results are presented in Table 1. The accuracy of all QCs was between −9.02 and 14.15%, and the intra- and inter-day CVs were <4.65 and 5.85%, respectively. All values of precision and accuracy were within the acceptance criteria, which demonstrated that the developed method was reproducible, precise, and accurate.

**Table 1:** Accuracy and precision results of KPT-335.

		5 ng/mL	10 ng/mL	400 ng/mL	800 ng/mL
Day1	Conc. mean (ng/mL)	5.13 ± 0.24	10.56 ± 0.26	413.55 ± 13.40	773.95 ± 18.03
	Accuracy	2.54	5.57	3.39	−3.26
	%CV	4.65	2.44	3.24	2.33
Day2	Conc. mean (ng/mL)	5.52 ± 0.06	10.97 ± 0.41	394.74 ± 10.80	767.19 ± 28.46
	Accuracy	10.48	9.69	−1.31	−4.1
	%CV	1.03	3.74	2.74	3.71
Day3	Conc. mean (ng/mL)	5.48 ± 0.09	10.9 ± 0.40	434.39 ± 9.35	856.32 ± 19.96
	Accuracy	9.59	9.00	8.60	7.04
	%CV	1.63	3.71	2.15	2.33
Inter-day	Conc. mean (ng/mL)	5.38 ± 0.23	10.81 ± 0.39	414.23 ± 19.76	799.15 ± 46.79
	Accuracy	7.54	8.09	3.56	−0.11
	%CV	4.30	3.60	4.77	5.85

Conc., Concentration; Accuracy = (actual concentration-theoretical concentration)/theoretical concentration*100%; Precision (%CV) = SD/ Conc.mean*100%; SD, standard deviation.

#### 3.1.4 Carryover

As shown in Figure 2D, the peak area responses of KPT-335 in the blank samples are <15.98% of the LLOQ responses and not more than 0.21% of the responses for the IS.

#### 3.1.5 Extraction recovery and matrix effect

The extraction recovery results are shown in Table 2. The average recoveries of LQC, MQC, and HQC samples of KPT-335 and IS were between 101.33 and 104.30% and the precision of KPT-335 and IS was between 1.32 and 5.65%, indicating that the concentration of the samples had no effect on the recovery of the analyte.

**Table 2:** Extraction recovery and matrix effect results of KPT-335.

Analytes	Concentration (ng/mL)	Extraction recovery (%)	%CV	Matrix effect (%)	%CV
KPT-335	10	101.33 ± 5.73	5.65	106.98 ± 9.95	9.3
	400	104.30 ± 4.97	4.77	/	/
	800	103.30 ± 1.36	1.32	99.95 ± 3.22	3.22
IS	400	103.19 ± 3.98	3.86	/	/

The presence of matrix had little effect on the determination of analytes. There was minimal variation in the matrix influencing factors between low and high concentrations of KPT-335. The average value of the normalized matrix factor and the precision of the six samples at low concentrations were 106.98 and 9.30%, respectively. The average value of the normalized matrix factor and the precision of the six samples were 99.95 and 3.22%, respectively. The detailed results are shown in Table 2.

#### 3.1.6 Dilution effect

The deviation in the accuracy of a single 10-fold dilution and three consecutive 10-fold dilutions were − 10.76 to 1.04% and − 8.54 to −0.91%, respectively, and the levels of precision were 4.70 and 2.86%, respectively.

#### 3.1.7 Stability

The stability results of KPT-335 in cat plasma or pure solution under the five different conditions are summarized in Table 3. Under all the tested conditions, the results were <±5% deviation from the nominal concentration, illustrating that KPT-335 offers favorable stability and is suitable for sample analysis.

**Table 3:** Stability results of KPT-335.

Stability	Concentration (ng/mL)	Recovery (%)	%CV
Room-temperature stability (2 h)	10	−12.06 ± 0.21	2.43
	400	−13.87 ± 3.58	1.04
	800	−8.04 ± 36.47	4.96
Freeze–thaw stability	10	5.40 ± 0.11	1.07
	400	1.14 ± 6.93	1.71
	800	−1.36 ± 21.97	2.78
Auto-sampler stability (24 h)	10	7.65 ± 0.36	3.36
	400	3.68 ± 4.47	1.08
	800	2.58 ± 16.01	1.95
Long-term stability (−20°C, 20 days)	10	6.87 ± 0.72	6.72
	400	−8.43 ± 14.29	3.90
	800	−5.57 ± 21.39	2.83
Stock solution stability (20 days)	10	−14.82 ± 2.87	9.00
	400	−5.84 ± 3.19	3.76
	800	−0.02 ± 3.29	1.49

The trial concluded that accuracy was not satisfactory after leaving the drug at room temperature in plasma for more than 2 h. Due to the short storage time at room temperature, we also investigated the effects of light and temperature on the drug in plasma. The sample recovery rate was between 59 and 67.5% after 8 h at room temperature. The recovery rate of the light-protected samples ranged from 60.4 to 63.2%. Sample recovery at 4°C was more than 80%. The results showed that light had almost no effect on the sample, whereas temperature had a greater effect. The lower the temperature, the longer the sample could be stored.

#### 3.1.8 Optimized room temperature stability

**Figure 3:**
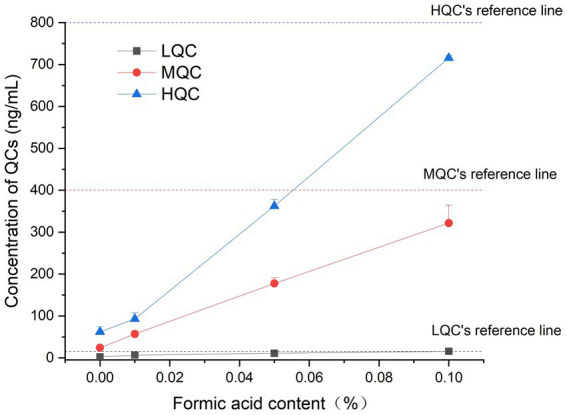
The concentration results of plasma samples containing different proportions of formic acid.

The results showed that when the formic acid content in the sample was 0.1%, the accuracy of the stable samples at room temperature could reach more than 85%, which met the requirements of the regulations, and confirmed that the plasma samples containing 0.1% formic acid could be stored at room temperature for 12 h. The results are shown in Figure 3.

#### 3.1.9 Reinjection reproducibility

Accuracy ranged from −11.06 to 13.69% for LQC samples, −11.41 to 3.25% for MQC samples, and − 12.01 to 3.14% for HQC samples. The 2-day results showed that the CV% of low, medium, and high concentration quality control sample were 6.84, 5.48, and 4.82%, respectively.

### 3.2 Pharmacokinetic study

**Figure 4:**
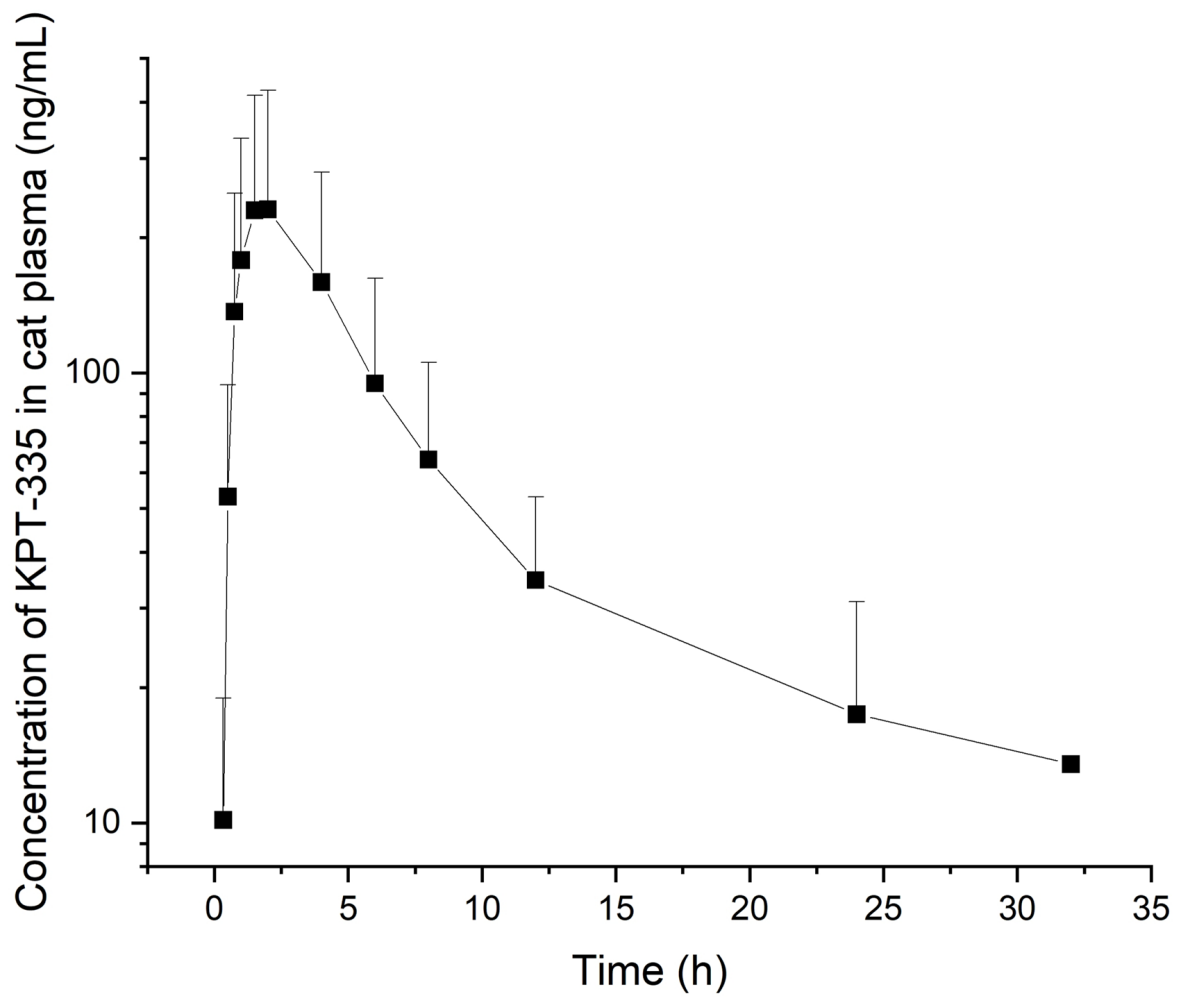
Mean plasma concentration-time profile of KPT-335 in cats after p.o.

After cats received a single oral dose of KPT-335 capsules at 2 mg/kg BW, the validated method was applied to the analysis of plasma samples obtained from cats. The average blood concentration was measured at different times and was plotted as a blood concentration-time curve as depicted in Figure 4. T_max_ was 1.46 ± 0.51 h; C_max_ was 239.54 ± 190.60 ng·mL^−1^; T_1/2_ was 5.16 ± 2.30 h; AUC_0-t_ was 1439.85 ± 964.64 ng·mL^−1^·h; AUC_0-∞_ was 1589.82 ± 1003.75 ng·mL^−1^·h. The remaining pharmacokinetic parameters are shown in Table 4. From the pharmacokinetic parameters, we can know that the drug is rapidly absorbed and eliminated in cats.

**Table 4:** Pharmacokinetic parameters of KPT-335 after p.o. administration in animal.

Animal species	Dose (mg/kg BW)	Parameters							
		Lambda__z_ (1/h)	HL__Lambda_z_ (h)	C_max_ (ng/mL)	T_max_ (h)	MRT_last_ (h)	AUC_last_ (h·ng/mL)	AUC_INF_obs_ (h·ng/mL)	AUC__%Extrap_obs_ (%)
Cat (*n* = 6)	2	0.15 ± 0.06	5.16 ± 2.30	239.54 ± 190.60	1.46 ± 0.51	5.46 ± 2.14	1439.85 ± 964.64	1589.82 ± 1003.75	11.32 ± 6.56
Dog (*n* = 6) (13)	1.5	/	3.88 ± 1.47	253 ± 88.3	3.83 ± 2.71	/	1760 ± 223	1810 ± 216	/
Dog (*n* = 4, per group) (12)	1.25	/	5	244.8	5.3	/	1576.5	/	/
	1.5	/	5	312	5.3	/	2346.8	/	/

Lambda__z_, Eliminate the rate constant; HL__Lambda_z_, Elimination phase half-life; C_max_, Peak concentration; T_max_，Peak time; MRT_last_, Mean residence time; AUC_last_, Area under the curve from the start of the administration time to the last point; AUC_INF_obs_, Area under the curve from the start of the administration time to the extrapolation to infinity; AUC__%Extrap_obs_, Area under the curve of the extrapolated part obtained after extrapolation to infinity accounts for the percentage of AUC_INF_obs_.

## 4 Discussion

### 4.1 Methodological establishment

Zhou et al. (16), Sauter et al. (17), and Li et al. (18) established a UPLC-MS/MS method for the detection of KPT-330 in rat or mouse plasma. The method used Acquity BEH C18 to separate KPT-330 and IS, 0.1% formic acid water and acetonitrile for mobile phases, and acetonitrile to precipitate proteins. By referring to the relevant literature on KPT-330, we established a UPLC-MS/MS method for detecting the concentration of KPT-335 in cat plasma. We experimented with positive and negative ion modes in the ion sweeping stage. We found that the signal-to-noise ratio and response in the positive ion mode was high, and the mobile phase in the positive ion mode was simpler, so the positive ion mode was selected. We compared the Acquity BEH C18 and Phenomenex Kinetex C18, and the Phenomenex Kinetex C18 was selected based on several parameters, including peak shape and retention time. Phases A and B used in the literature were selected as the mobile phases for this test. Acetonitrile has a stronger elution ability than methanol, and 0.1% formic acid was added to acetonitrile to improve the response of KPT-335. The mobile phase gradient was adjusted based on previous reports, and the gradient with the shorter peak time, the best KPT-335 correspondingly, and the least carryover was selected. The differences between the KPT-330 methodology in the literature and the KPT-335 methodology in this experiment are detailed in Table 5.

**Table 5:** Methodological differences compared.

Analytes	KPT-335	KPT-330(18)	KPT-330(16)	KPT-330(17)
Columns	Phenomenex Kinetex C18	Acquity BEH C18	Acquity BEH C18	Acquity BEH C18
Mobile phase	0.1% formic acid (A); 0.1% formic acid acetonitrile (B)	0.1% formic acid (A); acetonitrile (B)	0.1% formic acid (A); acetonitrile (B)	0.01% formic acid with 5% ACN (A); 0.01% formic acid acetonitrile (B)
Gradient	A:90% to 5–90%	A:90% to 10–90%	A:85% to 15–85%	A:70–40% to 2–70%
Flow rate	0.30 mL/min	0.40 mL/min	0.35 mL/min	0.50 mL/min
Column heater temperature	40°C	40°C	45°C	40°C
Sample processing	Acetonitrile:plasma 9:1	Acetonitrile:plasma 4:1	Acetonitrile:plasma 3:1	Acetonitrile:plasma 1:1

### 4.2 Methodological optimization

We selected 24 h for the initial investigation of room temperature stability, and the results were not within the requirement of ±15%. A second test was carried out, and the time was shortened to 8 h, but the accuracy still did not meet the requirement of ±15%. The results showed that higher concentrations led to a higher accuracy, and the accuracy was closer to the acceptable range when the stability time was shortened to 4 h. The storage time continued to be shortened, and the stability time at room temperature was set to 2 h in the last test. We found that the accuracy of all concentrations met the requirement of ±15%. In the room temperature stability optimization experiment, after adding formic acid, the drug was placed at room temperature in acidic plasma for 12 h, and its accuracy was still acceptable. We suspect that because the target is an alkaline drug and the pH value of the plasma is generally neutral or alkaline, the drug is in a molecular state under this condition and can aggregate easily. After adding acetonitrile, the aggregated molecules will precipitate with the protein, resulting in a decrease in the amount of free detectable drug. Adding a certain amount of formic acid to the plasma makes the plasma acidic. Consequently, the drug is in an ionic state and will not aggregate or be precipitated, and the accuracy can be satisfied.

### 4.3 Pharmacokinetic parameters

In a previous study, six healthy beagle dogs were administered KPT-335 at a single dose of 1.5 mg/kg BW after meals. It reached a peak concentration (C_max_) of 253 ± 88.3 ng/mL at 3.83 ± 2.71 h, with an area under the curve (AUC_0-t_) of 1760 ± 223 h·ng/mL, and a half-life (T_1/2_) of 3.88 ± 1.47 h (13). A comprehensive pharmacokinetic analysis was performed within 24 h of the administration of KPT-335 on day 14. When KPT-335 was administered at 1.5 mg/kg BW (four animals) and 1.25 mg/kg BW (four animals), C_max_ was 312 and 244.8 ng/mL, and AUC was 2346.8 and 1576.5 h·ng/mL, respectively, and the average T_max_ and T_1/2_ of all dogs were 5.3 and 5 h (12). There are no pharmacokinetic studies on KPT-335 in cats, and in our experiments, the T_1/2_ is 5.16 ± 2.30 h. We found that the half-life of KPT-335 in cats and dogs is relatively similar. According to Figure 3, there is a great variation between the concentrations of KPT-335 in the first few sampling points of six cats, resulting in a large difference in the overall pharmacokinetic parameters of the tested cats. This may be the result of fasting administration. Each test cat had different eating conditions before fasting, resulting in different gastrointestinal emptying and different gastrointestinal pH values. KPT-335 is greatly affected by pH and adopts a molecular form in a high-pH environment, which can aggregate and precipitate and may lead to large differences in drug absorption. If KPT-335 is administered after feeding, there is more gastric acid, the pH value in the environment is low, and the drug assumes an ionic state in the stomach and dissolves well. Therefore, the absorption of the drug is good until it gradually enters the intestine, and with the increase in pH, the drug gradually precipitates (21). Postprandial dosing may be considered and bioavailability will be assessed in a follow-up test.

## 5 Conclusion

In this study, we developed a rapid, simple, and accurate UPLC-MS/MS method for quantifying KPT-335 in cat plasma. The linearity, sensitivity, accuracy, and precision of the method met the relevant requirements. The method has a short pretreatment process, sufficient sensitivity, and a short elution time. Moreover, the validated method was successfully applied in a preliminary pharmacokinetic study of KPT-335 in cats. With the development of KPT-335 as a potential anti-tumor drug, this analytical method is suitable for further analysis in both non-clinical and clinical studies.

## Data availability statement

The original contributions presented in the study are included in the article/Supplementary material, further inquiries can be directed to the corresponding author.

## Ethics statement

The animal study was approved by the Experimental Animal Center of China Agricultural University (No. 11305-23-E-002). The study was conducted in accordance with the local legislation and institutional requirements.

## Author contributions

YY: Writing – original draft, Data curation, Methodology, Writing – review & editing. JQ: Methodology, Writing – review & editing. JK: Data curation, Writing – review & editing. YC: Data curation, Writing – review & editing. YL: Methodology, Writing – review & editing. SC: Methodology, Writing – review & editing. ZW: Methodology, Writing – review & editing. FS: Writing – review & editing. XC: Project administration, Supervision, Writing – review & editing.
