# Corrigendum: Development of a UPLC-MS/MS method for quantifying KPT-335 (Verdinexor) in feline plasma for a study of PK

**DOI:** 10.3389/fvets.2024.1486883

**Published:** 2024-09-23

**Authors:** Yuxin Yang, Jicheng Qiu, Jingyuan Kong, Yuying Cao, Yu Liu, Sumeng Chen, Zeyu Wen, Feifei Sun, Xingyuan Cao

**Affiliations:** ^1^Department of Veterinary Pharmacology and Toxicology, College of Veterinary Medicine, China Agricultural University, Beijing, China; ^2^College of Animal Science and Technology, Anhui Agricultural University, Hefei, China

**Keywords:** UPLC-MS/MS, KPT-335, pharmacokinetics, cat, plasma

In the published article, there was an error in Figure 1A as published.

When providing the structural formula, the wrong picture was uploaded.

The incorrect hydroxyl group was included where the original structural formula should feature the carbonyl group.

The corrected Figure 1 and its caption appear below.

**Figure 1:**
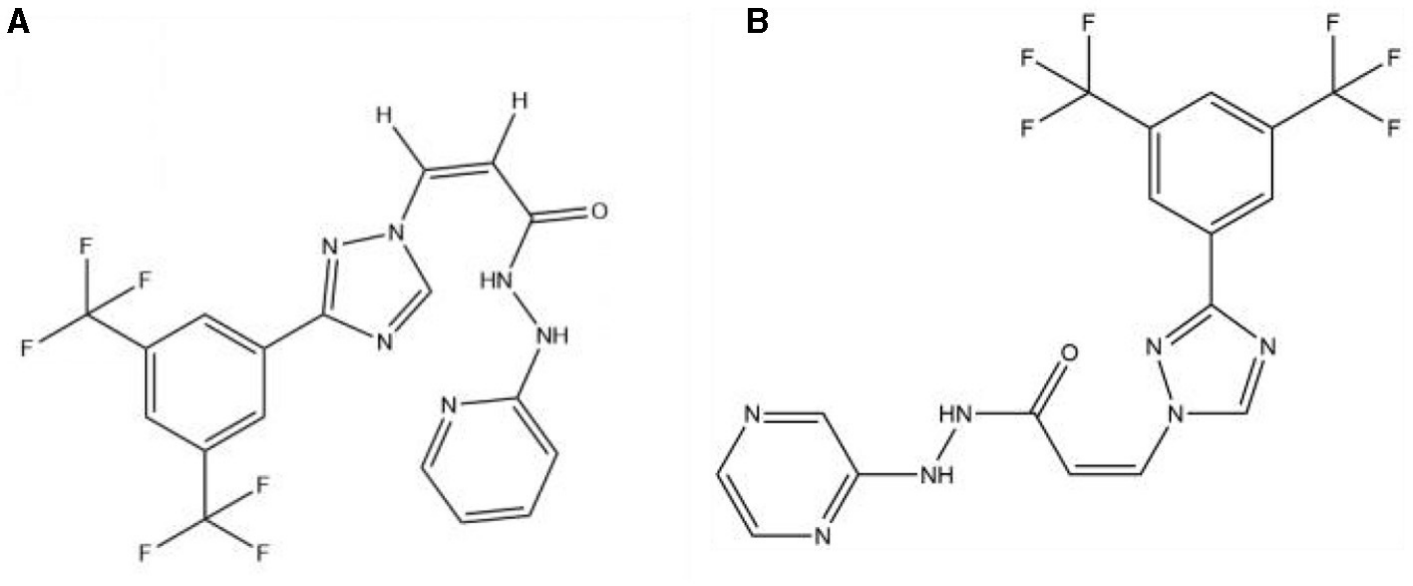
The structure of the KPT-335 **(A)** and KPT-330 **(B)**.

The authors apologize for this error and state that this does not change the scientific conclusions of the article in any way. The original article has been updated.

