# Supplementary material for: Development of a UPLC-MS/MS method for quantifying KPT-335 (Verdinexor) in feline plasma for a study of PK
Source: Front Vet Sci. 2024 Jul 26;11:1438295. doi: 10.3389/fvets.2024.1438295 (PMC11310113; doi:10.3389/fvets.2024.1438295)
Supplement: Supplementary file 1 [file Table_1.DOCX]

Supplementary Material

Table 1 Qualitative and quantitative ion pairs, tapered hole voltage and collision energy

| Drugs | Quantitative ion pair(*m/z*) | Qualitative ion pair (*m/z*) | Cone voltage(V) | Collision energy (eV) |
| --- | --- | --- | --- | --- |
| KPT-335 | 443.1＞334.0 | 443.1＞334.0 | 125 | 25 |
|  |  | 443.1＞110.1 |  | 25 |
| KPT-330 | 444.1＞334.0 | 444.1＞334.0 | 110 | 20 |
